# An unusual case of B-cell lymphoma of the scalp

**DOI:** 10.1093/jscr/rjad639

**Published:** 2023-11-29

**Authors:** Nadeem Chaudhry, Abid Qureshi, Sindhuri Gollamudi, Kinjal Kasbawala, Brittni J Clopton, Colton Moore, Romulo Genato, Philip Xiao, Armand Asarian

**Affiliations:** Department of Plastic Surgery, The Brooklyn Hospital Center, 121 Dekalb Ave, Brooklyn, NY 11201, United States; Department of Surgery, The Brooklyn Hospital Center, 121 Dekalb Ave, Brooklyn, NY 11201, United States; Department of Internal Medicine, The Brooklyn Hospital Center, 121 Dekalb Ave, Brooklyn, NY 11201, United States; Department of Surgery, The Brooklyn Hospital Center, 121 Dekalb Ave, Brooklyn, NY 11201, United States; Department of Surgery, The Brooklyn Hospital Center, 121 Dekalb Ave, Brooklyn, NY 11201, United States; Department of Surgery, The Brooklyn Hospital Center, 121 Dekalb Ave, Brooklyn, NY 11201, United States; Department of Surgery, The Brooklyn Hospital Center, Icahn School of Medicine at Mount Sinai, 121 Dekalb Ave, Brooklyn, NY 11201, United States; Department of Pathology, The Brooklyn Hospital Center, Icahn School of Medicine at Mount Sinai, 121 Dekalb Ave, Brooklyn, NY 11201, United States; Department of Surgery, The Brooklyn Hospital Center, Icahn School of Medicine at Mount Sinai, 121 Dekalb Ave, Brooklyn, NY 11201, United States

**Keywords:** lymphoma, non-Hodgkin, immune, malignancy, B-cell, PCFCL

## Abstract

B-cell lymphoma is a lymphoproliferative non-Hodgkin lymphoma arising from B cells, a type of immune lymphocytes that produces antibodies in the follicles of lymph nodes. Primary cutaneous B-cell lymphoma (PCBCL), a subtype of B cell lymphoma, originates within cutaneous tissue without evidence of extracutaneous involvement. There are very few reports of PCBCLs originating in the scalp. The most common tumors of the scalp are usually benign with only 1%–2% being malignant, most being basal cell carcinoma, squamous cell carcinoma, or melanoma. Primary cutaneous follicular cell lymphoma (PCFCL) is regarded as the most common lymphoma of the skin with an indolent course and favorable prognosis due to the response rate to treatment methods such as surgical removal with local radiotherapy, topical drugs, and intralesional therapies. This report highlights a rare case of PCFCL originating in the scalp, to raise awareness of a topic that requires continued established management.

## Introduction

B-cell lymphoma is a lymphoproliferative non-Hodgkin lymphoma arising from B cells, a type of immune lymphocytes that produces antibodies in the follicles of lymph nodes. Primary cutaneous B-cell lymphoma (PCBCL), a subtype of B cell lymphoma, originates within cutaneous tissue without evidence of extracutaneous involvement [1]. In 2018, the World Health Organization classified PCBCLs as a subtype of B-cell lymphomas, and currently makes up 20% to 25% of all primary cutaneous lymphomas. PCBCLs are further divided into three subtypes: primary cutaneous follicular cell (PCFCL), primary cutaneous marginal zone B-cell, and primary cutaneous diffuse large B-cell, leg type (PCDLBCL, LT) [2, 3]. Although PCBCLs can appear anywhere, there are very few reports of PCBCL originating in the scalp. Tumors of the scalp are usually benign with only 1%–2% being malignant; most being basal cell carcinoma, squamous cell carcinoma, or melanoma [4]. PCBCL can be a rare challenging diagnosis in the setting of the more prevalent malignancies, given its rare occurrence. In this report, we highlight a rare case of primary cutaneous follicular cell lymphoma originating in the scalp, to raise awareness of a topic that requires continued established management.

## Case report

A 56-year-old male, with a past medical history of myelodysplastic syndrome (MDS), hypertension, and chronic obstructive pulmonary disease, presented with a 4-month history of a progressively enlarging soft tissue mass on his scalp. He denied any associated pain or systemic symptoms of unexplained weight loss, fevers, itchy skin, and fatigue. Physical examination was remarkable for 2 cm × 2 cm right scalp nodularity. Nodularity was soft, non-tender, immobile, with mild erythematous changes. There was no evidence of lymphadenopathy, hepatosplenomegaly, or other lesions on the body. Due to the patient’s history of MDS, the lesion was suspected to be malignant. Subsequently, the patient underwent complete excision of the mass for further pathological analysis. Surgical pathology was positive for B-cell lymphoma of follicle center origin, suggestive of PCFCL. Due to the rarity of the diagnosis in the scalp, the specimen was sent to a tertiary facility for a second opinion, and the original diagnosis was reconfirmed. Patient underwent PET-CT for further staging and was remarkable for myeloproliferative changes throughout the bone marrow, splenomegaly, hypermetabolic right scalp nodules around the site of excision, hypermetabolic retroperitoneal lymphadenopathy, and axillary lymphadenopathy. Due to the extensive spread of lymphoma, the patient was started on chemotherapy with Rituximab. There were no postoperative complications.

### Pathology

**Figure 1:**
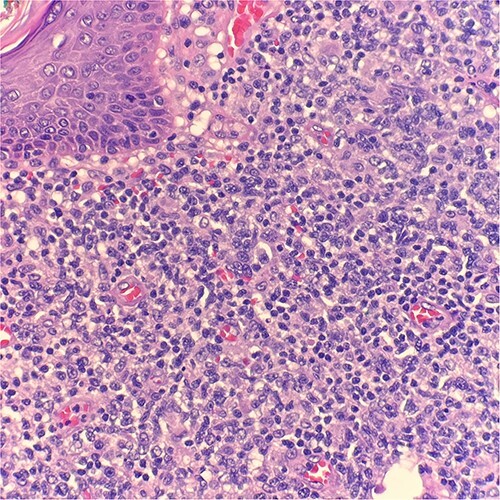
Microscopic examination reveals skin and subcutaneous tissue with atypical nodular nested lymphoid infiltrate in the dermis. H&E 40×.

**Figure 2:**
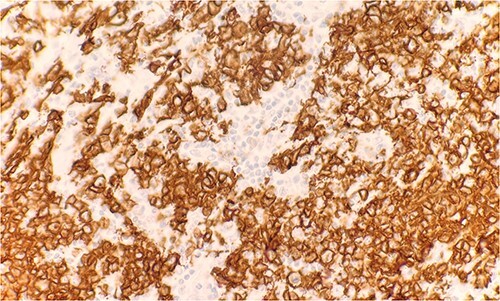
Immunohistochemical stain reveals atypical lymphoid cells are positive for CD20. IHC 40×.

**Figure 3:**
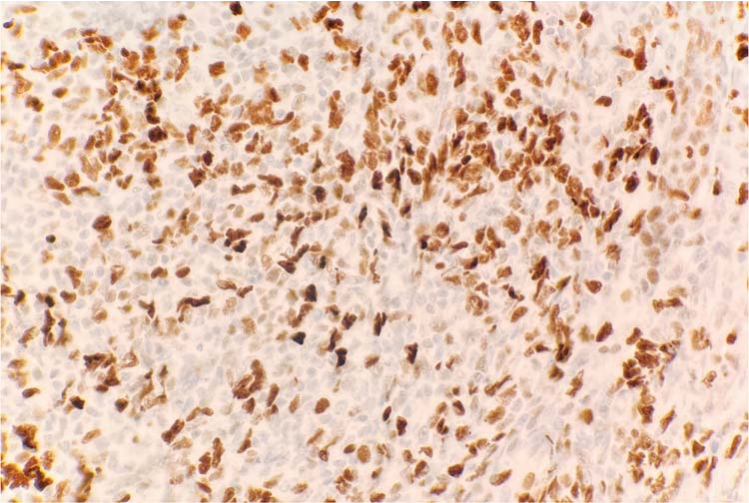
Immunohistochemical stain reveals atypical lymphoid cells are positive for BCL6. IHC 40×.

Microscopic examination reveals skin and subcutaneous tissue with atypical nodular nested lymphoid infiltrate in the dermis. The infiltrate is composed of intermediated cells with irregular nuclei, dispersed chromatin and variably conspicuous nucleoli mixed with large cells (Fig. 1). The atypical cells are positive for CD20 (Fig. 2) and BCL6 (Fig. 3). The morphological findings along with the skin location are consistent with primary cutaneous follicle center lymphoma (PCFCL).

## Discussion

PCFCL is regarded as the most common lymphoma of the skin with an indolent course and favorable diagnosis [5]. This cancer is predominantly found in Caucasian men, ages 50–60 yrs old and comprises the majority of PCBCLs at ~55%–60% [6, 7]. Lesions are commonly found on the head, neck, and trunk with some rare cases recently seen on the scalp. PCFCL can be identified with biopsy and immunohistochemical staining, FISH, and PCR. The commonly involved tumor markers are CD20+, CD79a+, CD5-, CD10+, and BCL 6+ [8–10]. Moreover, this disease has not been associated with the BCL2 locus translocation of t(14;18) which is a prognostic translocation observed with systemic follicular lymphoma. PET/CT or Pan CT scan should be performed at the time of diagnosis to evaluate for any systemic involvement along with lactate dehydrogenase, complete blood count with differential, and comprehensive metabolic panel per National Comprehensive Cancer Network guidelines [11].

PCFCL has a favorable prognosis due to the response rate to treatment methods such as surgical removal with local radiotherapy, topical drugs, and intralesional therapies [8]. PCFCL has a 5-year disease-specific survival rate of 95%, and cutaneous recurrence rate of 30%–46.5%. Patients present more commonly with a solitary lesion that is erythematous as either a papule, plaque, or nodule. These lesions are slow growing, often painless, and non-pruritic [6, 7, 9]. Treatment is often dependent on the symptoms experienced, location, and the tumor burden. For patients with many lesions spread throughout the upper extremities and trunk, low-dose local radiotherapy may be preferred. Radiotherapy has had an excellent response, however, can lead to alopecia scarring of the scalp [6]. For solitary lesions, surgical excision with negative margins may be executed but there is an associated increased rate of relapse in comparison to radiotherapy [12, 13].

Lesions too thick or numerous may not respond to radiotherapy for which Rituximab has been suggested as a first-line systemic agent [10]. Rituximab has been shown to be effective in extensive skin involvement and is subdivided as intravenous and intralesional injection. A prior study showed a 77% response rate when Rituximab was administered intravenously, with remission in 6–57 months and a recurrence rate of 20%; on the other hand, patients treated with intralesional injections had an 83%–89% response rate and recurrence rate of 40%–62%. These therapies have been reserved for patients who cannot undergo radiation therapy and surgical resection. Though recurrence rates are high, intralesional injection is more convenient and has been stated to have fewer adverse reactions, with most frequent reactions being urticaria and rash in 15% of patients and Steven–Johnson syndrome reported in <2% of cases [13, 14]. It should be standard guidelines that patients follow up every 6 months for a physical exam and continued monitoring of the treated lesions [6, 7]. Thus far, there have been no identifiable risk factors associated with this disease [9].

In conclusion, PCFCL is a rare form of B-cell lymphoma that often goes unnoticed due to the vast amount of benign tumors as well as other more commonly found malignancies, such as basal cell carcinoma or squamous cell carcinoma. Fortunately, this cancer has an excellent prognosis due to its response to multimodal therapies as well as a lack of systemic involvement.
